# Clinical and histopathological characteristics of *COL4A3* c.2881+1G>A variant causing Alport spectrum disorders in Croatian population

**DOI:** 10.17305/bjbms.2022.7567

**Published:** 2023-01-06

**Authors:** Matija Horaček, Tamara Nikuševa Martić, Petar Šenjug, Marija Šenjug Perica, Maja Oroz, Sania Kuzmac, Dragan Klarić, Merica Glavina Durdov, Marijan Saraga, Danko Milošević, Danica Batinić, Marijana Ćorić, Frane Paić, Danica Galešić Ljubanović

**Affiliations:** 1 Institute of Pathology, School of Medicine, University of Zagreb, Zagreb, Croatia; 2 Department of Medical Biology and Genetics, School of Medicine, University of Zagreb, Zagreb, Croatia; 3 Department of Nephropatology and Electron Microscopy, Dubrava University Hospital, Zagreb, Croatia; 4 Division of Rheumatology, Srebrnjak Children’s Hospital, Zagreb, Croatia; 5 Department of Gynecology and Obstetrics, Clinical Hospital “Sveti Duh”, Zagreb, Croatia; 6 Clinical Department of Pathology and Cytology, University Hospital Centre Zagreb, Zagreb, Croatia; 7 Department of Nephrology, General Hospital Zadar, Zadar, Croatia; 8 Department of Pathology, University Hospital Split, Split, Croatia; 9 Department of Pediatrics, University Hospital Split, Split, Croatia; 10 Department of Pediatrics, University Hospital Centre Zagreb, Zagreb, Croatia; 11 Pediatric ordination dr. Danica Batinić, Zagreb, Croatia; 12 Laboratory for Epigenetics and Molecular Medicine, School of Medicine, University of Zagreb, Zagreb, Croatia

**Keywords:** Alport syndrome (AS), thin basement membrane nephropathy (TBMN), proteinuria, collagen type IV, α3 chain of collagen IV, *COL4A3* c.2881+1G>A variant, targeted next-generation sequencing (NGS).

## Abstract

Alport syndrome (AS) and thin basement membrane nephropathy (TBMN) are part of the spectrum of kidney disorders caused by pathogenic variants in α3, α4, or α5 chains of the collagen type IV, the major structural component of the glomerular basement membrane (GBM). Using targeted next-generation sequencing (NGS), 34 AS/TBMN patients (58.8% male) from 12 unrelated families were found positive for heterozygous c.2881+1G>A variant of the *COL4A3* gene, which is considered disease-causing. All patients were from the continental or island part of Croatia. Clinical, laboratory, and histopathological data collected from the medical records were analyzed and compared to understand the clinical course and prognosis of the affected patients. At the time of biopsy or first clinical evaluation, the mean age was 31 years (median: 35 years; range: 1–72 years). Hematuria was present in 33 patients (97.1%) and 19 (55.9%) patients had proteinuria. There were 6 (17.6%) patients with hearing loss, 4 (11.8%) with ocular lesions, and 11 (32.4%) with hypertension. Twenty-three (67.6%) patients had proteinuria at follow-up, and five (14.7%) patients with the median age of 48 years (range: 27–55) progressed to kidney failure, started dialysis, or underwent kidney transplantation. Of the 13 patients who underwent kidney biopsy, 4 (30.8%) developed focal segmental glomerulosclerosis (FSGS), and 8 (66.7%) showed lamellation of the GBM, including all patients with FSGS. It is essential to conduct a detailed analysis of each collagen type IV genetic variant to optimize the prognosis and therapeutic approach for the affected patients.

## Introduction

Alport syndrome (AS) and thin basement membrane nephropathy (TBMN) are inherited renal disorders characterized by a structural defect of the glomerular basement membrane (GBM). The defect is caused by variants in the *COL4A3*, *COL4A4,* and *COL4A5* genes which encode α3, α4, or α5 polypeptide chains of collagen type IV (COL4), the main component of the human basement membrane in glomeruli, inner ear, and the eye [1–4].

The prevalence of AS is 1:5000 [5]. The most frequent cause of AS is a variant in the *COL4A5* gene (encoding α5 chain) located on the X chromosome, thus causing the X-linked type of AS (XAS). Autosomal recessive AS (ARAS) or autosomal dominant type of AS (ADAS) are caused by variants in the *COL4A3* or *COL4A4* genes (encoding α3 or α4 chains, respectively) [1, 6–13]. Analysis of COL4 variants showed that XAS occurs in about one in 2000 individuals and single heterozygous *COL4A3* and/or *COL4A4* variants in about one in 100 [14]. XAS, ARAS, ADAS, and double heterozygous (digenic) forms are the most common cause of inherited kidney disease and the second most common cause of inherited kidney failure (KF) [5].

In most cases, AS clinically presents with hematuria. Other characteristics are ultrastructural changes of GBM, sensorineural hearing loss, and ocular lesions [15]. In classic XAS, males are more severely affected than females. About 50% of males with XAS progress to KF by the age of 30, assuming no intervention [16, 17]. Male and female patients with ARAS have a similar clinical course to male patients with XAS, with KF occurring in the first or second decade of life. Although the ADAS is still a matter of literature debate, it shows high clinical variability, ranging from isolated hematuria to late-onset KF, sometimes associated with the development of focal segmental glomerulosclerosis (FSGS) [18–20]. In both ARAS and ADAS, males and females are equally affected. The rate of progression to KF and the presence of extrarenal symptoms depend on the localization and the type of the variant present. Progression to KF occurs more gradually in patients with ADAS than in those with ARAS or XAS [13, 21, 22]. The main ultrastructural characteristics of AS presented on electron microscopy (EM) are the thinning and thickening of the GBM with fragmentation, lamellation, tiny granules within the GBM, and the irregular outer contour of the GBM [15, 23].

Analysis of *COL4A3*, *COL4A4,* and *COL4A5* pathogenic variants in the Genome Aggregation Database (gnomAD; https://gnomad.broadinstitute.org) showed that the heterozygous *COL4A3* or *COL4A4* variants are present in about 1 in 100 individuals, making them the most frequent genetic kidney disorder [24]. Based on renal biopsy, heterozygous *COL4A3* and *COL4A4* patients are usually diagnosed with TBMN. Its primary clinical feature is persistent microscopic hematuria [4, 25], and the most common morphological finding of EM is diffuse thinning of the GBM [26]. A correlation between TBMN with heterozygous *COL4A3* and *COL4A4* variants and benign familial hematuria has been observed in several studies [27–31]. However, more recent studies have shown that not all heterozygous carriers of *COL4A3* and *COL4A4* variants had a benign course, with some individuals presenting with progressive hematuria and proteinuria, FSGS, hypertension, and KF [6, 9, 32–39]. Patients with the same *COL4* variant can have different clinical and histological findings, even in the same family [6]. For these reasons, Miner [2] introduced the term Alport spectrum disorder for all diseases caused by *COL4* gene variants. This term was discussed at the latest International Workshops on Alport Syndrome but is still under consideration by the wider community [40].

For the past 20 years, AS and TBMN research has been focused on the correlation of genotype and phenotype, describing the entire spectrum of phenotypes caused by variants in the *COL4* genes, with the location and type of variant largely determining the clinical course and disease prognosis [2, 16, 41, 42]. Recent advances in molecular pathology facilitated sequencing-based diagnostics for inherited kidney disease as the primary factor in making a definitive diagnosis and prognosis assessment. Notably, it is essential to accurately identify newly found variants and precisely determine their effect, role in the pathogenesis, and correlation with the clinical course for each patient. We can provide an adequate and early treatment and reliable prognosis with detailed and thoughtful analysis. Herein, we present clinical and histological features of the presumed founder variant c.2881+1G>A of the *COL4A3* gene identified in 12 families from the continental or island part of Croatia.

## Materials and methods

### Patients

This study was conducted as a part of the research project “Genotype–phenotype correlation in Alport’s syndrome and thin glomerular basement membrane nephropathy” funded by the Croatian Science Foundation (IP-2014-09-2151), approved by the Ethics Committee of the University of Zagreb School of Medicine (Number: 380-59-10106-15-168/181, Class: 641-01/15-02/01). All participants signed informed consent before blood sample collection and genetic testing. After genetic counseling, urinalysis, family members’ recruitment, and blood sampling for targeted next-generation sequencing (NGS), the heterozygous variant *COL4A3* c.2881+1G>A was detected in 39 participants from 12 unrelated families. Five patients declined to participate in the nephrological assessment and subsequent follow-up. Thus, they were excluded from the study. Out of 34 patients, 13 underwent kidney biopsy.

The patient demographic and clinical data (gender and age at diagnosis, the age of onset of the symptoms, presence of hematuria, proteinuria, hypertension, hearing loss, ocular abnormalities, the value of glomerular filtration rate, chronic kidney disease (CKD) stage, beginning of dialysis, transplantation, and follow-up data) were collected from medical records. We considered hematuria to be a finding of more than five red blood cells per high-power field (area visible under the microscope at 400× magnification). Proteinuria was defined as a positive result in the urine dipstick test or proteinuria higher than 150 mg in 24-h urine. The estimated glomerular filtration rate (eGFR) values were calculated using Chronic Kidney Disease Epidemiology Collaboration (CKD-EPI) equation (https://www.mdcalc.com/ckd-epi-equations-glomerular-filtration-rate-gfr) for adults and the Bedside Schwartz equation for children [43, 44]. CKD was defined as the abnormalities of kidney structure or function, present for >3 months, with an impact on health [45]. CKD stage 1 denotes maintained renal function (eGFR ≥ 90 ml/min/1.72 m^2^) with urinary abnormality (hematuria and proteinuria), stage 2 mildly decreased renal function (eGFR 60–89 ml/min/1.72 m^2^), stage 3a mildly to moderately decreased renal function (eGFR 45–59 ml/min/1.72 m^2^), stage 3b moderately to severely decreased renal function (eGFR 30–44 ml/min/1.72 m^2^), stage 4 severely decreased renal function (eGFR = 15–29 ml/min/1.72 m^2^), and stage 5 (eGFR < 15 ml/min/1.72 m^2^) denotes KF. A correlation of clinical and histopathological data and a genealogy study was also performed.

### Histopathological analysis

Kidney biopsy samples were routinely analyzed by light microscopy (hematoxylin-eosin, Periodic Acid-Schiff, Masson trichrome, and Jones methenamine silver stain), immunofluorescent analysis (IgG, IgA, IgM, C3, C1q, kappa, and lambda light chains), and EM. The stages of interstitial fibrosis and tubular atrophy (IFTA) were defined as mild (stage 1), affecting less than 25% of the specimen; moderate (stage 2), affecting 25%–50%; and severe (stage 3), affecting more than 50% of the specimen. In all participants (except one patient without glomeruli in tissue prepared for EM), the GBM thickness was measured on EM images using iTEM software (Olympus Soft Imaging Solutions GmbH). Altogether three measurements of the GBM thickness per capillary loop on ten random capillary loops per specimen were made (i.e., 30 measurements per patient) using a modified direct method of measuring GBM thickness as previously described by Haas [1]. Furthermore, the distance between the podocyte and endothelium cell membrane was measured with a predefined threshold for the thin GBM according to the standardized method of GBM thickness measurements established at the Department of Nephropatology and Electron Microscopy, Dubrava University Hospital [46].

### DNA isolation, PCR-based library preparation, and NGS

The genomic DNA was isolated from peripheral blood samples using Zymo Quick-DNA Miniprep Plus Kit (Zymo Research, Orange, CA, USA), following the manufacturer’s procedure. DNA concentration was measured using a High Sensitivity Qubit quantification kit (Life Technologies, Carlsbad, CA, USA).

Libraries with amplicon sizes ranging from 125 to 175 base pairs (bp) were prepared following the manufacturer’s procedure using AmpliSeq Library Plus and AmpliSeq Custom DNA Panel (Illumina, San Diego, CA, USA), which includes two primer pools with 89 and 87 primer pairs, respectively, that can amplify all coding and flanking splice regions of *COL4A3*, *COL4A4*, and *COL4A5* genes. A 20 ng of genomic DNA was used to amplify the targeted genes in a single-tube multiplex reaction. Adapter sequences and molecular indexes were incorporated during PCR steps, and amplicon libraries were purified using magnetic beads Agencourt AMPure XP reagent (Beckman Coulter, Brea, CA, USA). Library quality was assessed with Agilent Bioanalyzer HS DNA Kit (Agilent Technologies, Santa Clara, CA, USA), showing adequate sample sizes and concentrations. Libraries were then quantified using a High Sensitivity Qubit quantification kit (Life Technologies, Carlsbad, CA, USA) and pooled to generate a sequencing library with a final loading concentration of 50 pM.

Subsequent Illumina sequencing was carried out on the Illumina iSeq 100 System platform (Illumina, San Diego, CA, USA) with Standard flow cell following the manufacturer’s instructions. The mean coverage depth of all amplicons achieved by targeted NGS for all samples was 270×. Sequencing generated the corresponding FASTQ files, and bioinformatical analysis was performed using the Illumina VariantStudio software (Germline workflow; version 2.12.0.34) (Illumina, San Diego, CA, USA).

### Sanger sequencing

The splice donor variant c.2881+1G>A found in *COL4A3* at genomic position chr2: 228149062 (variant described according to reference genome GRCh37) was confirmed with standard dye-terminator sequencing. Genomic DNA was amplified using forward (FW) 5’ GGG GGA ACA AGG AGA TAA AGG A3’ and reverse (RV) 5’ AAA CAC TGG CCC TCA CTG TC3’ primers and EmeraldAmp MaX HS PCR Master Mix (Takara, Berkley, CA, USA). PCR products were purified using ExoSAP-ITTM PCR Product Cleanup Reagent (Thermo Fisher Scientific, Waltham, MA, USA). Sanger sequencing was performed on the ABI310 (Applied Biosystems, Foster City, CA, USA) with BigDye v1.1 chemistry (Thermo Fisher Scientific, Waltham, MA, USA). Results were visualized with Vector NTI software (Thermo Fisher Scientific, Waltham, MA, USA).

### Variant classification

The described *COL4A3* variant c.2881+1G>A (LRG_230t1:c.2881 +1G>A) located at the genomic position chr2:228149062 (GRCh37.p13) affects the splice-donor sequence of intron 34 of *COL4A3* gene potentially leading to exon skipping. We detected no other variants in *COL4A3, COL4A4,* or *COL4A5* genes. However, we cannot exclude additional genetic and nongenetic factors which may contribute to disease severity. The c.2881+1G>A variant was not found in the GnomAD database (https://gnomad.broadinstitute.org). However, the variant was reported in two patients (one male and one female) from Germany in Leiden Open Variant Database (LOVD) and described as pathogenic, causing ADAS (https://databases.lovd.nl/shared/variants/in_gene?search_geneid=%3D%22COL4A3%22&search_VariantOnTranscript/DNA=%3D%22c.2881%2B1G%3EA%22). There were no additional clinical or histopathological data in those reports. Furthermore, *in silico* bioinformatics tools, VarSEAK (https://varseak.bio/) and Mutation Taster (http://www.mutationtaster.org) predicted disease-causing effects. Nevertheless, the expected damaging effect of c.2881+1G>A should be confirmed with functional studies.

The Gharavi Laboratory at Columbia University reported variant c.2881+1G>T in the *COL4A3* gene at the same position but with a different nucleotide substitution and classified it as pathogenic (https://clinvarminer.genetics.utah.edu/submissions-by-variant/NM000091.4%28COL4A3%29%3Ac.2881%2B1G%3ET).

According to our knowledge, *COL4A3* c.2881+1G>A variant could be considered a founder variant as it was detected with high frequency in a homogeneous and closed community on the island Pašman, Croatia. Guidelines of joint consensus recommendation of the American College of Medical Genetics and Genomics and the Association for Molecular Pathology [47] and Recommendations for interpreting the loss of function PVS1 ACMG/AMP variant criterion [48] were followed during the interpretation of the variant.

### Statistical analysis

Descriptive statistical analysis methods were used to evaluate demographic and clinical data. Mean, median, standard deviation (SD), and interquartile range (IQR) were calculated for numeric variables, when appropriate. Frequency values were used to describe categorical data. Statistical analyses of data were performed using Fisher’s exact test, unpaired independent-samples *t*-test, Mann–Whitney U test, and Kruskal–Wallis test, when appropriate. The occurrence of adverse events (onset of KF, dialysis, or kidney transplantation) was calculated utilizing the Kaplan–Meier method and compared between groups with the log-rank test. The hazard ratio (HR) for adverse events was estimated by Cox regression analysis. As Alport spectrum disorders are genetic disorders and disease onset data retrieved during patients’ interviews are subjective, we decided to mark the starting age in the analysis of renal survival time (i.e., time to an adverse event or last follow-up) as 0 years. All data were analyzed using IBM SPSS Statistics for Windows v.29.0 (IBM Corp., Armonk, NY, USA). All statistical tests were two-sided, and intergroup differences with *P* < 0.05 were considered significant.

## Results

### Variant data

Atotal of 39 participants from 12 unrelated families reported positive for heterozygous variant *COL4A3* c.2881+1G>A at genomic position chr2:228149062 (Table 1). There were no previous descriptions of this variant in The Human Gene Mutation Database (HGMD) and Ensembl genome database. Bioinformatic analysis showed PVS1, PM2, and PP3 levels of certainty for pathogenicity by ACMG [47]. The described variant was a null variant (within ± 2 of canonical splice site) affecting *COL4A3*, which is a known mechanism of disease (gene has 221 known pathogenic variants), associated with ADAS and ARAS (PVS1). The variant was not found in GnomAD exomes (coverage 61.8%) nor GnomAD genomes (coverage 30.3%) despite good coverage (PM2). The variant has a pathogenic computational verdict based on four pathogenic predictions from DANN, EIGEN, FATHMM-MKL, and MutationTaster versus no benign predictions. *In silico* software results were DANN score: 0.9936; EIGEN prediction: Pathogenic (raw scoring 1.1059; prediction coding score 17.7662); FATHMM-MKL prediction: Damaging (coding score 0.989); MutationTaster prediction: Disease-causing (probability 1). The comparison of an in-house database of 50 healthy individuals tested for variants in *COL4A3, COL4A4*, and *COL4A5* genes also support our analysis results. We suspect *COL4A3* c.2881+1G>A to be a founder variant as it was detected with high frequency in a homogeneous and closed community on the Pašman island in Croatia. It is also a pathogenic variant according to *in silico* analysis and the fact that it is located in the non-coding splice-site locus.

**TABLE 1:** *COL4A3* c.2881+1G>A variant data

**VARIANT DATA**						
**Gene**	**Zygosity**	**Consequence**	**HGVS nomenclature**	**Genomic position**	**Exon / Intron**	**Clinical significance**
***COL4A3***	Heterozygous	Splice donor	c.2881+1G>A	Chr2: 228149062	Intron 34-35	Pathogenic

HGVS: Human Genome Variation Society

### Clinical data

The baseline (at the time of biopsy or first clinical evaluation) clinical features of 34 out of 39 positively tested patients (5 of them declined to participate in further nephrological follow-up), with proven *COL4A3* c.2881+1G>A variant, are shown in Table 2. The cohort included 20 (58.8%) men and 14 (41.2%) women, with a mean age at the onset of symptoms (deciphered from patient interview) of 16.69 ± 14.32 years (range: 3 months–55 years). At the time of biopsy or first clinical evaluation, the mean age was 31.0 ± 20.5 years (range: 1–72 years). Hematuria was present in 33 participants (97.1%); 5 (14.7%) of them had macrohematuria, whereas 19 (55.9%) patients had proteinuria. One male patient (H05, 3-years old) without hematuria or proteinuria and with maintained renal function was tested as a member of a family with confirmed *COL4A3* c.2881+1G>A variant and was also positive. The hearing loss was detected in 6 (17.6%) patients, with 4 of them being members of the same family (three patients had confirmed sensorineural hearing loss on an audiogram). Ocular abnormalities were detected in 4 (11.8%) patients, whereas 11 (32.4%) patients presented with hypertension. The mean eGFR at the time of biopsy or first clinical evaluation was 85.9 ml/min/1.72 m^2^ (median: 91.5 ml/min/1.72 m^2^; range: 10–144.3 ml/min/1.72 m^2^). According to eGFR, 20 (58.8%) patients had stage 1 CKD, 8 (23.5%) had stage 2, 3 (8.8%) had stage 3b, 2 (5.9%) had stage 4, and 1 (2.9%) patient (D11) had stage 5 CKD (Table 2). One (2.9%) patient (I01) with CKD stage 4 underwent dialysis at that time. Altogether, at the time of biopsy or the first clinical evaluation, 2 (5.9%) patients reached stage 5 CKD or started dialysis.

**TABLE 2:** Patients’ baseline (at the time of biopsy or first clinical evaluation) demographic and clinical data

**Code**	**Sex**	**Age of**	**Age at**	**Hematuria**	**Proteinuria**	**24-h**	**eGFR**	**CKD**	**Hearing**	**Ocular**	**Hypertension**
		**onset**	**diagnosis**			**proteinuria**	**(ml/min/1.73 m^2^)**	**stage**	**loss**	**abnormalities**	
		**(years)**	**(years)**			**(g/dU)**					
A02	F	3.0	47.0	Yes	Yes	2.86	69	2	No	No	Yes
A04	M	16.0	45.0	Yes	Yes	3.14	31	3b	No	No	Yes
B01	M	15.0	15.0	Macro	Yes	0.30	120.2	1	No	No	No
C01	F	55.0	55.0	Yes	Yes	N/A	21.0	4	No	No	No
C02	M	20.0	35.0	Macro	Yes	<0.08	117.0	1	No	No	Yes
C03	M	4.0	4.0	Yes	No	<0.08	70.2	2	No	No	No
C04	F	6.0	6.0	Yes	No	<0.08	70.0	2	No	No	No
D02*	M	1.0	1.0	Macro	No	<0.08	68.5	2	No	No	No
D04	F	3.0	40.0	Yes	Yes	1.46	97	1	Yes	No	No
D06	F	48.0	48.0	Yes	Yes	1.96	34.0	3b	Yes	Yes	Yes
D07	M	3.0	38.0	Yes	Yes	4.64	37	3b	Yes	No	No
D11	F	22.0	27.0	Yes	Yes	1.20	10.0	5	Yes	Yes	Yes
E01	F	32.0	42.0	Yes	Yes	0.46	102.6	1	No	No	No
E03	M	2.5	2.5	Yes	No	<0.08	95.7	1	No	No	No
E04	F	4.0	4.0	Yes	No	<0.08	118.6	1	No	No	No
E05	M	13.0	13.0	Yes	No	<0.08	144.3	1	No	No	No
E07	M	9.0	9.0	Yes	Yes	0.20	86.2	2	No	No	No
F01	M	5.0	16.0	Yes	No	<0.08	81.8	2	No	No	No
H01	M	19.0	37.0	Yes	No	<0.08	120.0	1	No	No	No
H02	F	7.0	11.0	Yes	No	<0.08	119.1	1	No	No	No
H03	F	35.0	61.0	Yes	Yes	1.00	67.0	2	Yes	Yes	Yes
H05	M	0.3	3.0	No	No	<0.08	128.5	1**	No	No	No
I01	M	8.0	46.0	Yes	Yes	2.90	29.0	4	No	No	Yes
I02	F	6.0	15.0	Yes	No	<0.08	128.5	1	No	No	No
J02	F	15.0	35.0	Yes	No	<0.08	89.0	2	No	Yes	No
J03	F	11.5	11.5	Macro	No	<0.08	130.2	1	No	No	No
K01	M	11.0	34.0	Yes	No	<0.08	96.0	1	No	No	No
K02	M	25.0	65.0	Yes	Yes	N/A	71.0	2	No	No	Yes
K04	M	22.0	36.0	Yes	No	<0.08	92.0	1	No	No	No
K05	M	30.0	62.0	Macro	Yes	2.04	93.0	1	No	No	Yes
K06	M	32.0	72.0	Yes	Yes	N/A	91.0	1	No	No	Yes
K07	M	18.0	30.0	Yes	Yes	N/A	119.0	1	No	No	No
L01	M	47.0	48.5	Yes	Yes	1.09	70.0	2	Yes	No	Yes
M01	F	19.0	40.0	Yes	Yes	0.23	104.0	1	No	No	No

M: male; F: female; dU: daily urine; eGFR: estimated glomerular filtration rate; CKD: chronic kidney disease; *Patient D02 was born with unilateral renal agenesis; **included in CKD stage 1 because of the presence of a variant.

The follow-up data (median follow-up age was 37.3 years; range: 4.5–72 years), as shown in Table 3, revealed that three more patients (A04, C01, and D06) reached CKD stage 5, started dialysis, or underwent kidney transplantation. The median age of patients with adverse events (onset of KF, dialysis, or kidney transplantation) (*n* = 5; 14.7%) at the follow-up was 48 years (IQR: 41.3–54.3 years), with the youngest patient having 27 and the oldest 55 years. Hematuria was present in 30 patients (88.2%), whereas 6 (17.6%) had macrohematuria. Follow-up data also showed that four more patients developed proteinuria (23 in total; 67.6%). The mean eGFR on follow-up was 78.9 ml/min/1.72 m^2^ (median: 89.3 ml/min/1.72 m^2^; range: 4–150.5 ml/min/1.72 m^2^). Furthermore, according to calculated eGFR values, 17 (50%) patients had stage 1 CKD, 9 (26.5%) had stage 2 CKD, 2 (5.9%) had stage 3b, 4 (11.8%) had stage 4, and 2 (5.9%) patients had stage 5 CKD (Table 3).

**TABLE 3:** Patients’ demographic and clinical data at follow-up

**Code**	**Sex**	**Age at**	**Hematuria**	**Proteinuria**	**24-h**	**eGFR**	**CKD**	**Dialysis or**	**Age at**
		**follow-up**			**proteinuria**	**(ml/min/1.73 m^2^)**	**stage**	**transplantation**	**the start**
		**(years)**			**(g/dU)**				**of D/Tx**
A02	F	55.0	Yes	Yes	1.96	24.0	4	No	
A04	M	54.0	Yes	Yes	<0.08	24.0	4	Yes	54
B01	M	40.0	No	Yes	0.90	121.0	1	No	
C01	F	59.0	Yes	Yes	1.00	4.0	5	Yes	55
C02	M	40.0	Macro	Yes	0.45	113.0	1	No	
C03	M	14.0	No	Yes	N/A	114.5	1	No	
C04	F	19.0	Yes	Yes	0.30	98.0	1	No	
D02*	M	6.0	No	No	<0.08	89.3	2	No	
D04	F	44.0	Yes	Yes	3.58	81.0	2	No	
D06	F	72.0	Yes	Yes	2.15	15.0	4	Yes	48
D07	M	38.5	Yes	Yes	8.31	35	3b	No	
D11	F	32.0	Yes	Yes	<0.08	6.0	5	Yes	27
E01	F	54.0	Yes	Yes	0.37	89.2	2	No	
E03	M	16.0	Yes	No	<0.08	97.7	1	No	
E04	F	13.0	Yes	No	<0.08	112.1	1	No	
E05	M	15.0	Yes	No	<0.08	96.8	1	No	
E07	M	11.0	Yes	No	<0.08	108.1	1	No	
F01	M	17.0	Yes	No	<0.08	80.8	2	No	
H01	M	34.0	Macro	Yes	0.55	100.0	1	No	
H02	F	13.0	Yes	No	<0.08	normal**	1	No	
H03	F	70.0	Yes	Yes	0.48	44.0	3b	No	
H05	M	4.5	No	No	<0.08	normal**	1	No	
I01	M	46.0	Yes	Yes	2.90	18.0	4	Yes	46
I02	F	15.0	Macro	Yes	0.43	150.5	1	No	
J02	F	54.0	Yes	No	<0.08	69.0	2	No	
J03	F	19.5	Yes	Yes	0.59	113.9	1	No	
K01	M	34.0	Yes	Yes	0.22	115.0	1	No	
K02	M	66.0	Yes	Yes	0.25	77.0	2	No	
K04	M	36.0	Macro	No	<0.08	113.0	1	No	
K05	M	61.5	Macro	Yes	1.85	73.0	2	No	
K06	M	72.0	Yes	Yes	N/A	70.0	2	No	
K07	M	30.0	Yes	No	<0.08	115.0	1	No	
L01	M	56.0	Yes	Yes	2.14	64.0	2	No	
M01	F	61.0	Macro	Yes	0.54	94.0	1	No	

M: male; F: female; dU: daily urine; eGFR: estimated glomerular filtration rate; CKD: chronic kidney disease; D: dialysis; Tx: transplantation; *Patient D02 was born with unilateral renal agenesis; ** in medical records, eGFR was marked as normal without accurate numerical data.

**Figure 1.**
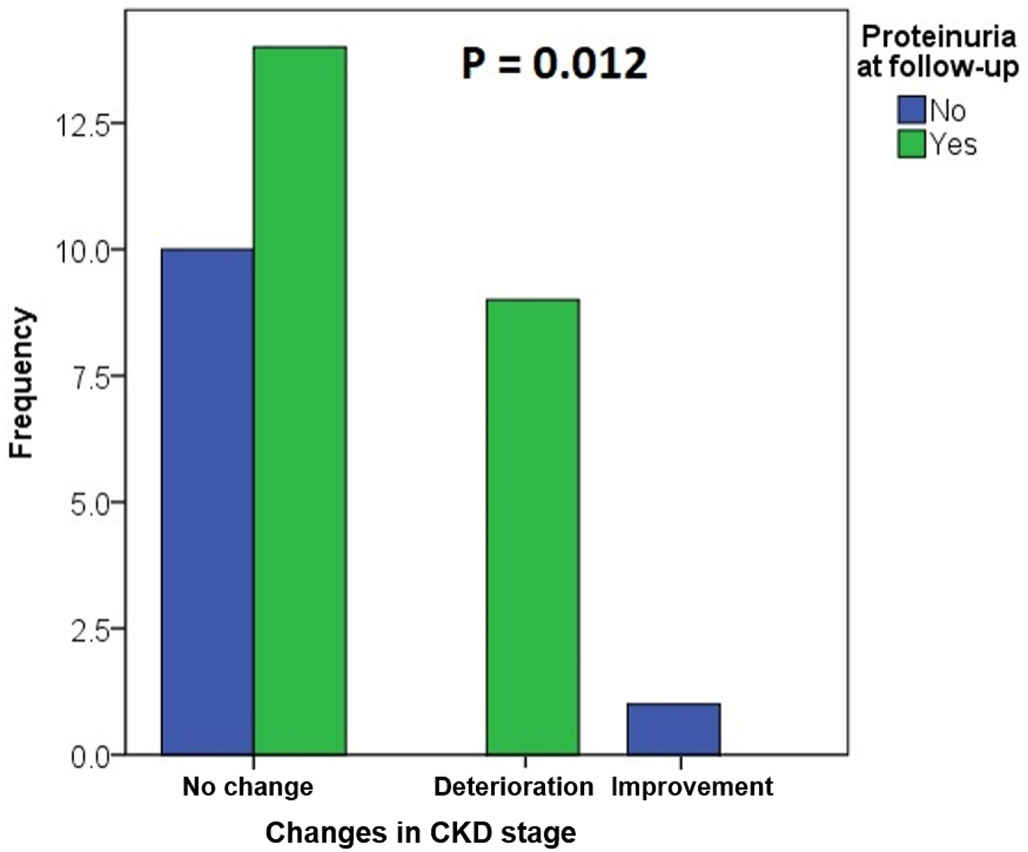
**Proteinuria and changes of the CKD stage between the time of diagnosis and an adverse outcome or the last follow-up.** All patients with a deterioration of the CKD stage had proteinuria (*P* = 0.012 between patients with proteinuria and without proteinuria, Fisher’s exact test). CKD: chronic kidney disease

By comparison with baseline patients’ characteristics in 24 patients (70.6%) at follow-up, the CKD stage was the same; only 1 (2.9%) patient experienced an improvement, whereas the CKD stage worsened in 9 (26.5%) patients. There is a statistically significant difference (*P* = 0.012) between patients without proteinuria (11; 32.4%), where none had deterioration of the CKD stage, and patients with proteinuria (23; 67.6%), where 9 (26.5%) showed a deterioration of the CKD stage (Figure 1).

**Figure 2.**
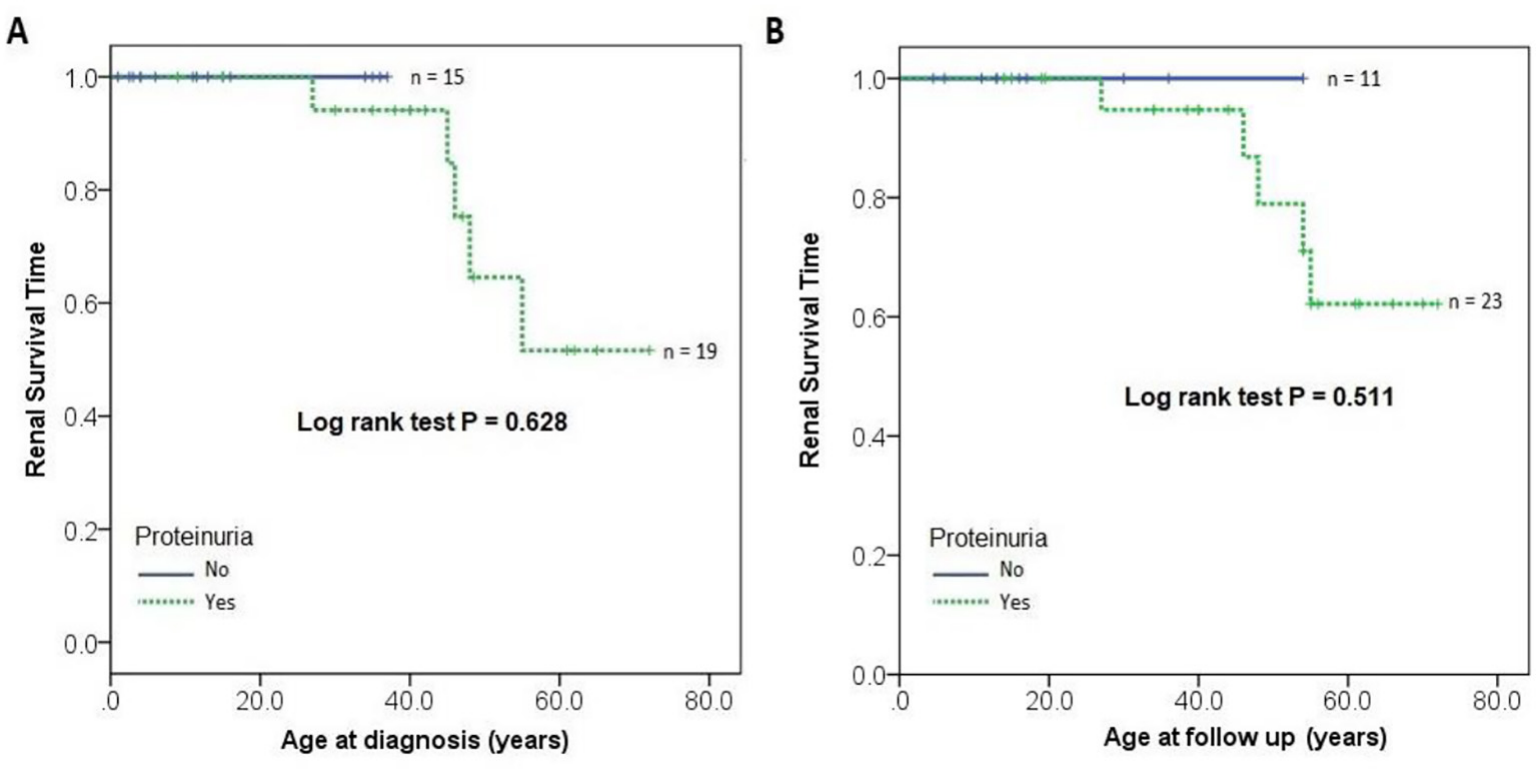
**Renal survival time in relation to proteinuria at diagnosis or follow-up.** The Kaplan–Meier method and the log-rank statistic for time to an adverse event (onset of KF, dialysis, or kidney transplantation) showed no statistically significant association with proteinuria at the time of diagnosis (A) or the last follow-up (B). KF: kidney failure.

Although the Kaplan–Meier method and the log-rank statistics for time to an adverse event (onset of KF, dialysis, or kidney transplantation) showed no statistically significant association with proteinuria (Figure 2), Cox regression analysis showed that patients with proteinuria at baseline or follow-up had a higher, although not statistically significant, the tendency (increased HR) toward the adverse renal outcome than the patients without proteinuria (basal: HR = 27.86, *P* = 0.762; follow-up: HR = 23.6; *P* = 0.637).

### Histopathological data

**Figure 3.**
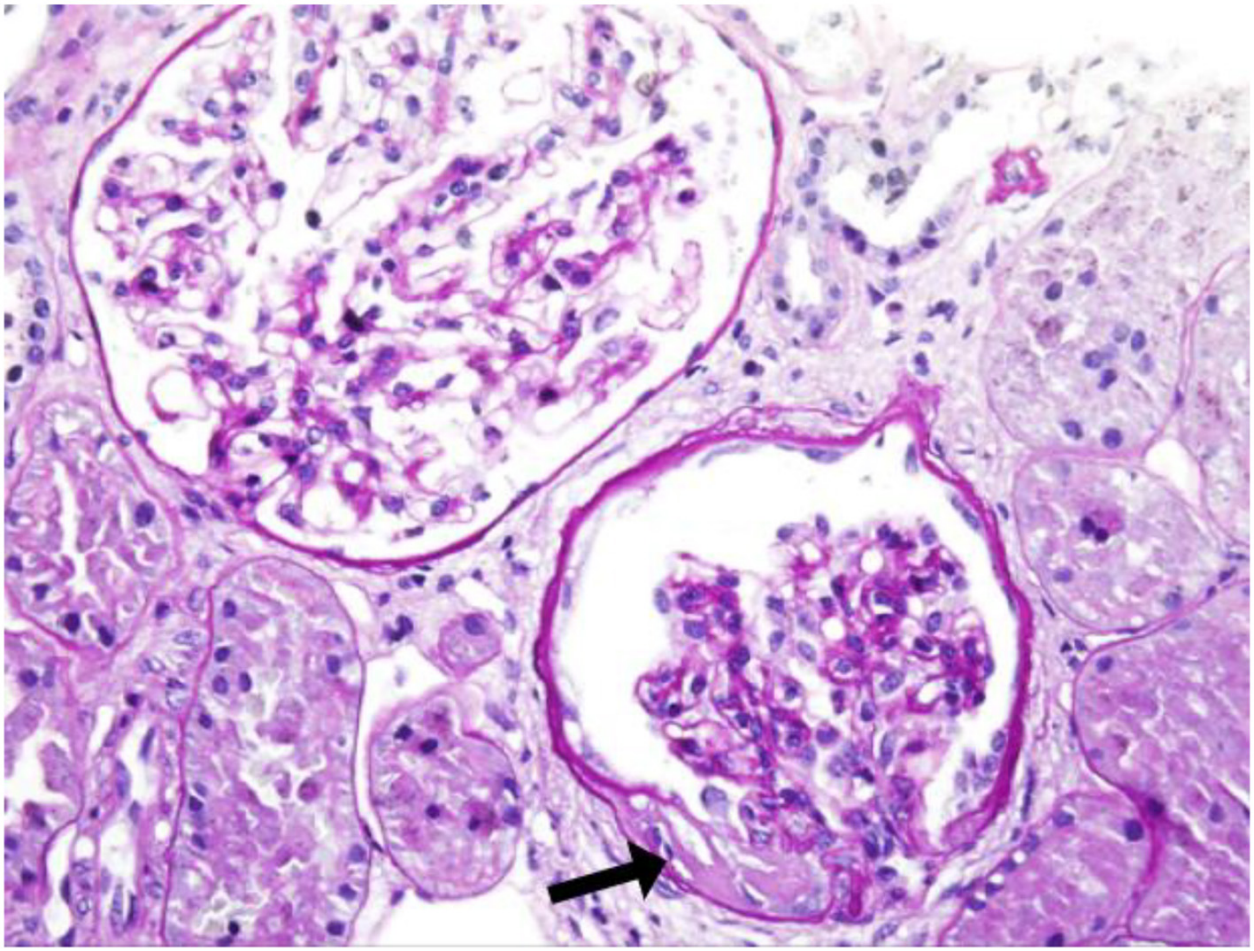
**Kidney biopsy specimen of patient D04 on light microscopy.** Perihilar focal segmental glomerulosclerosis (black arrow) in one glomerulus. Other 18 glomeruli were enlarged and without other morphological changes. There was no interstitial fibrosis or tubular atrophy. Arteries and arterioles had normal morphology. PAS stain, original magnification ×400.

Kidney biopsy data were available for 13 patients (Table 4). Histopathological diagnoses were: 8 (61.5%) with TBMN, 2 (15.4%) with AS, and 2 (15.4%) with TBMN and FSGS. One patient (7.7%) had advanced chronic changes with a high percentage of global and segmental glomerular sclerosis and prominent IFTA, but no glomeruli were available for EM. FSGS was found in 4 (30.8%) specimens, all of which were perihilar type (Figure 3). IFTA was mild (stage 1) in the majority of patients (86.4%). Only one patient (7.7%) had severe (stage 3) and one (7.7%) moderate (stage 2) IFTA. Fibrointimal thickening of the arterial wall was found in seven (53.8%) patients and arteriolosclerosis was present in five (38.5%) patients.

**TABLE 4:** Kidney biopsy data

**Code**	**Sex**	**Age at**	**Histopathological**	**FSGS**	**IFTA**	**Arteriolosclerosis**	**Fibrointimal**	**Average GBM**	**Lamellation**	**Alteration of**
		**biopsy**	**diagnosis**		**stage**		**arterial**	**thickness**		**thinning and**
		**(years)**					**thickening**	**(nm)**		**thickening of GBM**
A02	F	47.0	TBMN + FSGS	Yes	1	No	Yes	154	Focal	Yes
A04	M	45.0	Severe sclerosing lesions	Yes	3	Yes	Yes	No glomeruli on EM		
B01	M	15.0	TBMN	No	1	No	No	136	Focal	Yes
D04	F	40.0	AS	Yes	1	No	No	210	Yes	Yes
D07	M	38.0	TBMN + FSGS	Yes	2	Yes	Yes	252	Focal	Yes
E01	F	42.0	TBMN	No	1	No	Yes	158	No	No
E03	M	2.5	TBMN	No	1	No	No	170	No	No
F01	M	16.0	TBMN	No	1	No	No	246	No	No
H03	F	61.0	AS	No	1	Yes	Yes	263	Yes	Yes
J02	F	35.0	TBMN	No	1	No	No	226	Focal	Yes
J03	F	11.5	TBMN	No	1	No	No	217	Focal	Yes
K01	M	34.0	TBMN	No	1	Yes	Yes	172	Focal	Yes
L01	M	48.5	TBMN	No	1	Yes	Yes	252	No	Yes

M: male; F: female; AS: Alport syndrome; TBMN: thin basement membrane nephropathy; FSGS: focal segmental glomerulosclerosis; IFTA: interstitial fibrosis and tubular atrophy; GBM: glomerular basement membrane; EM: electron microscopy

**Figure 4.**
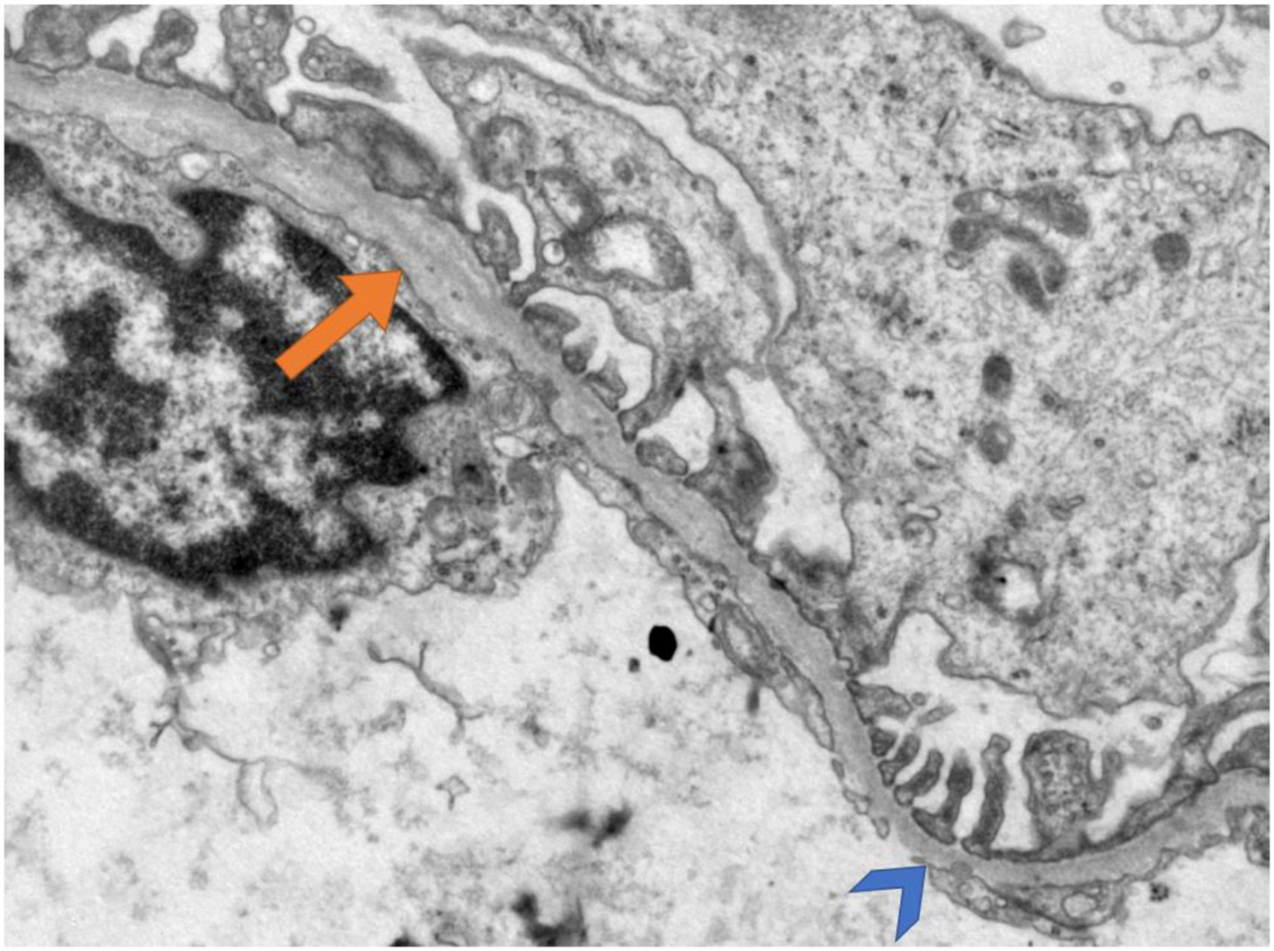
**Kidney biopsy specimen of patient J03 on electron microscopy.** Thinning of glomerular basement membrane (arrowhead) with focal lamellation (arrow). Transmission electron microscopy, original magnification ×15.000.

As there were no glomeruli in one specimen for EM, the ultrastructural analysis was conducted for 12 patients. The average GBM thickness was 204.7 nm (median thickness: 213.5 nm; range: 72–699 nm). Lamellation was present in eight (66.7%) cases; in six (50.0%) was only focally present (Figure 4).

## Discussion

**Figure 5.**
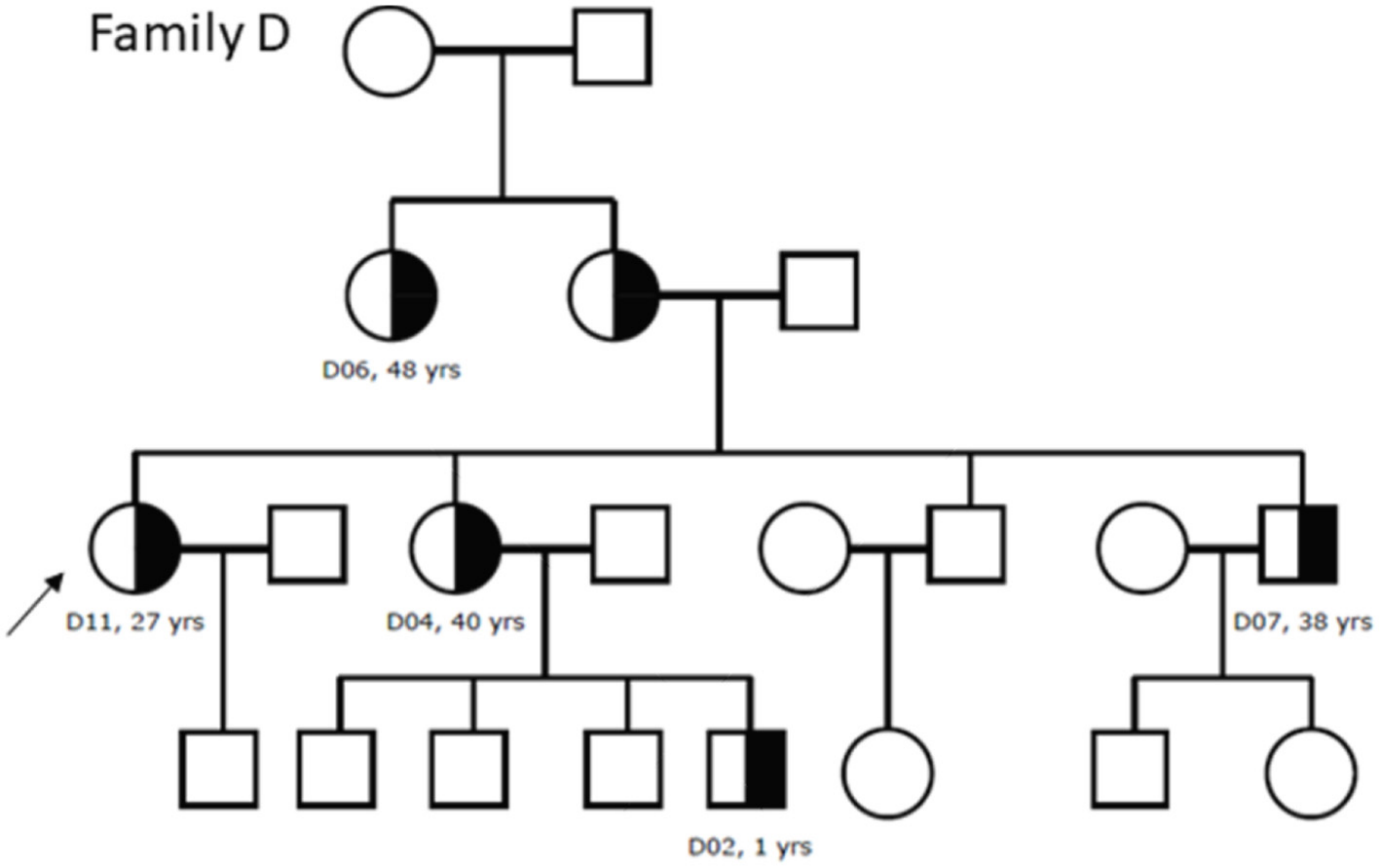
**Family pedigree of family D. The affected heterozygous members are marked with black half and probands are marked with an arrow.** We had no data for the eldest family members. All patients had hematuria (D02 had macrohematuria) and all except D02 had proteinuria. D04 had maintained renal function (CKD stage 1), D02 had CKD stage 2, D06 and D07 had CKD stage 3b, and D11 had kidney failure (CKD stage 5). Patients D04, D06, D07, and D11 had hearing loss, whereas D06 and D11 had ocular abnormalities and hypertension. Patient D04 was diagnosed with Alport syndrome with lamellation on electron microscopy and focal segmental glomerulosclerosis on light microscopy. Patient D07 was diagnosed with thin basement membrane nephropathy and focal segmental glomerulosclerosis with only discrete focal lamellation found on electron microscopy.

**Figure 6.**
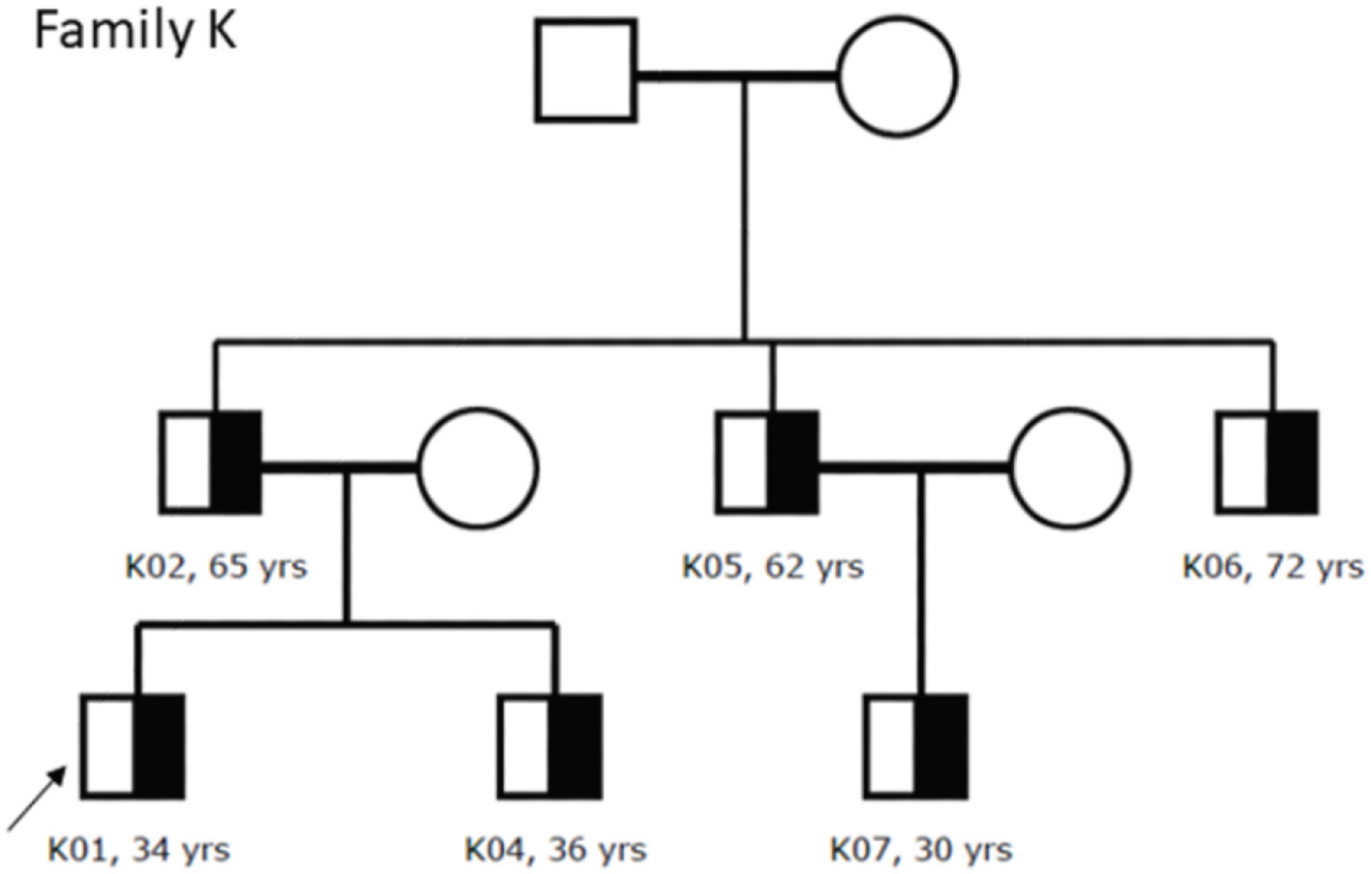
**Family pedigree of family K. The affected heterozygous members are marked with black half and probands are marked with an arrow.** We had no data for the eldest family members. All patients had hematuria (K05 had macrohematuria) whereas K02, K05, K06, and K07 had proteinuria. Patient K02 had CKD stage 2 whereas others had maintained renal function (CKD stage 1). None had hearing or ocular abnormalities. Patients K02, K05, and K06 had hypertension. Patient K01 was diagnosed with thin basement membrane nephropathy with only discrete focal lamellation on electron microscopy.

A total of 39 participants from 12 unrelated families reported positive for the heterozygous variant *COL4A3* c.2881+1G>A detected with NGS. After collecting clinical and histopathological data from the participants and obtaining the NGS results, we performed data correlation and genealogy study, generating family pedigrees (Figures 5 and 6).

Even though the age of onset was not consistent, our results showed that this variant causes disease, with half of the patients being under 14 years of age when the first symptoms manifested themselves. Renal symptoms were predominant. Hematuria was present in almost every patient and proteinuria in 55.9%. Extrarenal symptoms affected 20.6% of patients. Follow-up data showed that 14.7% of patients progressed to KF, started dialysis, or underwent kidney transplantation by the median age of 48 years. FSGS was found in 30.8% of patients with a kidney biopsy. The main predictive factor was proteinuria (Figure 1), as its occurrence indicates a more severe phenotype and is also an indication for starting treatment with ACE inhibitors [8, 33].

Several studies showed that *COL4* variants cause a wide range of renal disorders, including classical XAS, ARAS, and hematuria due to TBMN, whether hematuria is the first and only symptom for the rest of the individual’s life or it progresses to KF due to the development of FSGS [6, 34, 37, 49, 50]. Such substantial variability in phenotype, especially among patients with heterozygous *COL4A3* and *COL4A4* variants, is sometimes present even within the same families [3]. In our cohort, this variability was present both in clinical and histopathological findings, especially among members of family D (Tables 2 and 3). We have previously described similar clinical and histopathological variability in the pathogenic variant *COL4A4* c.193-2A> C [51].

*COL4* (*COL4A3, COL4A4,* and *COL4A5*) variants were also identified in patients with familial FSGS, broadening the spectrum of structural disorders of the GBM [35, 52–54]. Although TBMN, caused by autosomal *COL4* variants, has been considered a benign disorder with an excellent prognosis, several publications convincingly show that this is inaccurate [6, 9, 33]. These studies indicate that some patients develop proteinuria and FSGS and progress to KF, raising the question of ADAS naming [34].

In his research in 2014, Miner [2] stated that *COL4* variants, with clinically distinct but similar ultrastructural manifestations, were better understood as a spectrum of AS. This would cover all disorders from benign familial hematuria (with no consequent effect on renal function) to classical AS with undetectable COL4 in the GBM, eventual progression to KF, auditory and visual disturbances, and everything in between these two extremes. Given that a variant that causes hematuria in one person can cause progressive kidney disease in another, Miner [2] also stated that classifying these disorders into specific parts of the AS spectrum would be helpful for clinicians, as well as for patients and their families, to become familiar with the true risk of kidney disease. In 2021, at the latest International Workshops on Alport Syndrome [40], the term AS spectrum disorder was accepted to refer to all disorders caused by variants in the *COL4A3*, *COL4A4,* or *COL4A5* genes.

In 2020, in their systematic literature review of heterozygous *COL4A3* and *COL4A4* variants, Matthaiou et al. [55] presented clinical data as follows: hematuria was present in 94.8% of patients and proteinuria in 46.4%. Of all patients, 15.1% developed KF by the median age of 52.8 years (age range 21–84 years). Hearing loss was reported in 15.6% and ocular abnormalities in 3% [55]. On light microscopy, FSGS was present in 39.9%, and on EM, they found lamellation of the GBM in 6.9% of cases [55]. In comparison, our data considering the specific variant *COL4A3* c.2881+1G>A showed that at the time of biopsy or first clinical evaluation, hematuria was present in 97.1%, proteinuria in 55.9%, hearing loss in 17.6%, and ocular abnormalities in 11.8%, whereas at the time of the last follow-up, 30 (88.2%) patients had hematuria, 23 (67.6%) had proteinuria, and 5 (14.7%) developed KF, started dialysis, or underwent kidney transplantation by the median age of 48 years (age range 27–55 years). Even though we would expect all patients to have hematuria, three of them had hematuria at the time of biopsy or first clinical evaluation and no hematuria on follow-up while one patient had no hematuria at all. These facts highlight the importance of regular follow-up due to intermittent hematuria which is described in patients with TBMN [56]. FSGS was found on 30.8% of biopsies, and an interesting finding is that in our cohort, lamellation of the GBM (including discrete focal lamellation) was present in 66.7% of patients.

The phenotypic spectrum of *COL4* variants has been expanded to include disorders from isolated hematuria due to TBMN to more severe phenotypes, such as ARAS, ADAS, and FSGS, which can progress to KF. One hypothesis is that the spectrum of disorders caused by *COL4* variants is a multifactorial disorder whose phenotypic expression depends on the interaction of pathogenic variants affecting primary genes, modifying genes, and environmental factors [2, 6, 8, 33, 34, 37, 49].

However, there are two limitations of our study considering the additional molecular analysis. For now, we are unable to conduct the sequencing of other genes associated with hematuric nephritis and genetic FSGS and to analyze additional genetic modifiers that aggravate severe phenotypes. Also, some variants in *COL4* genes, such as deep intronic variants that may contribute to disease severity, cannot be detected with NGS [57].

## Conclusion

The suspected founder variant *COL4A3* c.2881+1G>A is disease-causing. These patients differ not only in clinical presentation but also in histopathological findings. It is characterized by hematuria and expressed extrarenal symptoms in 20.6% of patients and potential risk of developing FSGS and progression to KF in 14.7% of affected individuals. Proteinuria should be considered as an indicator of a potential decline in renal function since 26.5% of patients with proteinuria showed a decline in renal function whereas all patients without proteinuria had maintained renal function between the time of kidney biopsy and last follow-up.

Alport spectrum disorders caused by *COL4A3*, *COL4A4*, and *COL4A5* variants should always be considered potentially serious and, therefore, a detailed and constructed clinical, histopathological, and genetic assessment of each patient should be performed. An appropriate therapeutic and prognostic approach should be adjusted for each individual according to the severity of the clinical course, morphological changes, and type of variant detected with genetic analysis.

**Conflicts of interest:** Authors declare no conflicts of interest.

**Funding:** This research was conducted as part of the research project “Genotype-phenotype correlation in Alport’s syndrome and thin glomerular basement membrane nephropathy,” funded by the Croatian Science Foundation (IP-2014-09-2151).
